# Clonal diversity and spatial dissemination of multi-antibiotics resistant *Staphylococcus aureus* pathotypes in Southwest Nigeria

**DOI:** 10.1371/journal.pone.0247013

**Published:** 2021-02-23

**Authors:** Akinniyi Paul Akinduti, Joshua Adekunle Osiyemi, Temitope Temitayo Banjo, Oluwaseun Ejilude, Maged El-Ashker, Adewale Gideon Adeyemi, Yemisi Dorcas Obafemi, Patrick Omoregie Isibor

**Affiliations:** 1 Microbiology Unit, Department of Biological Sciences, Covenant University, Ota, Nigeria; 2 Department of Microbiology, University of Ilorin, Ilorin, Nigeria; 3 Department of Microbiology, Crawford University, Igbesa, Ogun State, Nigeria; 4 Microbiology Laboratory, Sacred Heart Hospital, Lantoro, Abeokuta, Nigeria; 5 Department of Internal Medicine and Infectious Diseases, Faculty of Veterinary Medicine, Mansoura University, Mansoura, Egypt; 6 Department of Civil Engineering, Covenant University, Ota, Nigeria; Instituto de Technologia Quimica e Biologica, PORTUGAL

## Abstract

Spread of genetically diverse *Staphylococcus aureus* characterized with multi-antibiotic resistance and regulated by high level *agr* functionalities in several communities in southwest Nigeria was investigated and evaluated for infection control. *Staphylococcus aureus* pathotypes recovered from 256 cases including purulent pus from skin infections, soft tissue aspirates, wounds, otorrhea, eye, throat and endocervical infections were assayed for biofilm and antibiogram. Further genotyped with micro-array, mapped for geospatial distribution and evaluated for clonal diversity and functional accessory gene regulators (*agr*). Significant Staphylococci infection among the ages (OR:0.021, CI:0.545–1.914) and female gender with prevalence rate of MSSA (53.0%) and MRSA (1.5%) (OR:1.021, CI:0.374–1.785) were observed. More than 52.5% resistance rates to tetracycline and amoxicillin with significant median resistance were observed in all the infection cases (p = 0.001). Resistance rate of 78.8% at MIC_50_ 32μg/ml and MIC_90_ 128μg/ml to amoxicillin-clavulanate, and more than 40% resistance to ceftazidime, ciprofloxacin and tetracycline of MIC_90_ and MIC_50_ at 32 μg/ml were observed. Strains with multi-antibiotic resistance index above 0.83, high beta-lactamase and strong biofilm clustered into separate phylo-group. Heterogeneous t442 (wound and pus), t657 (wound), t091 (ear) and t657 (ear and wound) revealed high phylogenetic diversity. Only 4.6% *pvl*+ MSSA-CC1 *agr*I, *pvl*+ MSSA-CC5 (13.6%) and *pvl*+ MRSA-CC7 *agr*II (4.6%), expressed enterotoxin, leukocidins, proteases and resistance gene determinants. Livestock clonal types clustered with identified community-associated strains. Clonal dissemination of resistant *pvl+* MSSA-CC1 and MRSA-CC5 encoding *agr* were predominant in several peri-urban communities where adequate geno-surveillance, population-target antimicrobial stewardship, extensive community structured infection control programs are needed to prevent further focal dissemination.

## Introduction

Staphylococcal infection remains a major health challenge in several countries, with huge resultant adverse effect ranging to life-threatening diseases such as pneumonia, bacteremia to high mortality cases [1]. Several clonal complexes have been reported from different regions of the globe [2]. Various *spa* types kept evolving with diverse genomic recombination, phylogenetic clones, and repeated nucleotide mutations, giving rise to fatal virulent strains [3]. In addition, there is a capability of numerous clonal strains of *Staphylococcus aureus* to adapt by its specificity for colonization through production of poly-N-acetylglucosamine to produce biofilm needed to evade immune response and antibiotic activity [4].

Severity of staphylococci infection correlates with virulence expression which is regulated through the functionality of accessory gene regulators (*agr)*, which encodes a two-component signal transduction system that could down-regulate surface proteins metabolism and up-regulate secreted proteins during *in vitro* growth [5], favoring the transcription of several secreted virulence factors (particularly enterotoxins, hemolysins and Toxic shock syndrome toxin-1) [6]. Functional *agr* groups were reported to enhance persistent staphylococci bacteraemia and soft tissue tropism with low antibiotic susceptibility to penicillin, cephalosporin and vancomycin [7,8]. Similar clonal spread of MRSA (methicillin-resistant *Staphylococcus aureus*) and MSSA (methicillin-susceptible *Staphylococcus aureus*) is becoming pandemic in several communities in Africa, mostly Nigeria where animal husbandry, behavioural responses and declined demographic factors enhance continuous dissemination of staphylococcal infection with high degree of antibiotic resistance [9]. The misuse and unregulated prescription of penicillin derivatives in high and uncontrollable proportion for treating several extra-intestinal infections such as abscess, ear infections, subcutaneous tissue inflammation, nasal discharges particularly in children and post-surgical wound culminated in a high rate of resistance and continuous development of methicillin-resistance strains [10,11].

Heterogenous *spa* types identified among several MSSA and MRSA carriers and infected subjects [12] require clonal diversity and staphylococcal infection surveillance, tracking and strains genotyping [13,14]. Moreover, evolution of various *spa* types has kept driving dynamics spread of staphylococcal infection that were demonstrated in various infection outbreaks, localized epidemics and community-acquired infections. Mapping the spread and dissemination of *mecA* gene among *spa* types is highly needed for reliable genomic tracking, localization and control of staphylococcal infection in several local communities with high-level dissemination and distribution of resistant *spa* types probably acquired from livestock [12].

In this study, we investigated the antibiotic resistance distribution and prevalence of *agr* groups of phylo-diverse *S*. *aureus* strains characterized by various *spa* repeats and assessed the potential association between different *agr* group functionalities, clonal diversity and staphylococci infection controls.

## Methods

### Isolates collection

Non-repetitive clinical samples totaling 256 including purulent pus (n = 58), skin aspirates and effusions (n = 34), wounds (n = 55), otorrhea (n = 36), eye infection (n = 14), throat (n = 35) and endocervical (n = 24), collected between June 2017 and August 2018 from overall 12,654 outpatients. This included patients from neighbouring states attending three major health facilities which serve as referral clinics in southwest Nigeria. Ethical permission for the study was granted by the Federal Medical Centre Abeokuta Health Research Ethics Committees with protocol approval: FMCA/470/HREC/09/2017; NHREC/08/10-2015 with permission from other health facilities but data on their gender, age, disease conditions and subjects’ location of residence were not fully retrieved. Each sample were cultured for Staphylococci strains and phenotypically characterized on Baird-Parker agar and Mannitol salt agars, Gram stained for cellular morphology, tested for catalase and coagulase production as previously discussed [15].

### Phenotypical beta-lactamase detection and antibiogram

Beta-lactamase production was assayed with modified starch-acidometric method [16] and Minimum inhibitory concentrations (MICs) for each antibiotic class against the strain was determined using micro-broth dilution assay [17] with 12 panel antibiotics consisting of tetracycline (TE), ceftazidime (CAZ), ciprofloxacin (CIP), gentamycin (GEN), amoxycillin-clavulanic acid (AMC), cefuroxime (CRO), ofloxacin (OFX), sulfamethoxazole (SXT), erythromycin (E), fosfomycin (FOX), vancomycin (VA) and Linezolid (LZD). Phenotypic resistance was interpreted according to CLSI guidelines [18]. Phenotypic screening for methicillin resistance was further determined by assessment of Staphylococcal growth on Mannitol salt agar and Mannitol salt agar supplemented with 4μg/ml Oxacillin as previously described [19]. Multi-antibiotic resistance index (MARI) was determined for each isolates.

### Biofilm detection

Phenotypic assessment of biofilm production was done in micro-broth bioassay [20]. Briefly, overnight pure colonies were suspended in 200μl Brain Heart infusion (BHI) broth supplemented with 0.25% glucose and incubated at 37C for 24 hours. After incubation, the growth medium was aspirated away and the wells were washed thrice with Phosphate Buffer Saline (PBS). The wells were stained with 0.5% w/v Crystal Violet for 5 minutes and gently rinsed. Ethanol (70%) was added to dissolve the Crystal Violet and absorbance of the stained biofilm solution was measured with UV Spectrophotometer against absorbance of non-biofilm producing *Staphylococcus aureus*.

### *mecA* and *pvl* genotyping

For detection of pathogenic MRSA, isolates were genotyped for *mecA* and *pvl* as described by Acevedo et al [21]. Briefly, DNA template was extracted using simple boiling method [22] and amplification was performed in separate reaction volume of 20 μl containing 0.8 μl of 10uM each primer of *mec5* (AAAATCGATGGTAAAGGTTGGC) and *mec6* (AGTTCTGCAGTACCGGATTTGC) and *pvl* gene with primers *pvl*-F (AATGAAATGTTTTTAGGCTCAAGACA) and *pvl*-R (TGGATAACACTGGCATTTTGTGA) with DNA template (1μl) and water (18 μl) for each reaction following previous described multiplex protocol [21]. Amplification reaction was carried out at initial denaturation of 94°C for 5 min, and followed by 30 cycles of denaturation 94°C for 45 s, annealing 60°C for 60 s, elongation 72°C for 1 min and final elongation 72°C for 5 min. Amplicon of 10 μl of each reaction was electrophoresed on 2% agarose TBE gel at 100V along with the marker. Bands of each PCR products were analyzed regarding their presence and size by using the positive control and the marker as references. MARI (multi-antibiotic resistance index) was determined by dividing the number of resisted antibiotics with total number of antibiotics used to which the organism was subjected. Relatedness of the MARI pattern, degree of biofilm production, beta-lactamase production and mecA detection among the identified strains from various sources were evaluated with dendrogram analysis constructed with DendroUPGMA algorithm.

### Genotyping and clonal diversity of *spa* types

Extracted genomic DNA obtained from overnight culture, was typed for *S*. *aureus* protein A (*spa* gene). PCR assay was performed in constituted reaction mixture of 2x MyTaq HS Mix (10μL), containing *spa* primers; *spa*1095F (5’-AGACGATCCTCCGGTGAGC-3’), *spa*1517R (5’-GCTTTTGCAATGTCATTTACTG-3’) of 5μL each and 1μL template DNA through 30 cycles of denaturation at 94°C for 30 seconds, annealing at 60°C for 30seconds and elongation at 72°C for 30 seconds, with final extension at 72°C for 5minutes [23,24]. DNA of *S*. *aureus* DSM 1104L strain served as a positive and distilled water as negative control. Quality of amplicon products was examined on electrophoresed agarose gel and positive strains were purified with GFX PCR DNA and Gel Band Purification Kit (GE Healthcare). Purified PCR products were sequenced with forward primer *spa*1095F using BigDye 3.1 terminator sequencing and analyzed on ABI Genetic Analyzer 3500Dx (Applied Biosystems, CA, USA). Categorisation of *spa* types was carried out with Based Upon Repeat Pattern (BURP) algorithm of the Ridom Staph Type software version 1.4 (RidomGmbH, Sedanstr, Germany) to cluster all *spa* types in the database according to *spa* clonal complexes [25]. Clonal diversity of Nigerian *spa* types with other meta-spa sequences were analysed with MEGA software (version 6.0).

### Virulence and resistance genotyping

Encoded *S*. *aureus* strains with *mecA* and *pvl* were further genotyped with StaphyType DNA microarray (Alere Technologies GmbH, Jena, Germany) for other virulence genes. Approximately 170 distinct genes and their allelic variants were targeted for PCR amplification and hybridization on Microtiter strip-mounted DNA microarrays following manufacturer’s instruction and the image of the array was recorded and analysed using a designated reader and software (Arraymate, Iconoclust, Alere Technologies) [26].

### Geospatial analysis

Geographical coordinates of individual subjects with staphylococcal infection were identified and recorded with differential global positioning system (GIS) and interpolated for analysis in ArcGIS 10.5.1 programme with respect to land division according to boundary marks in southwest Nigeria [27].

### Data analysis

To identify variables and risk factors that could influence staphylococcal infection rate among dependent variables (age, gender and clinical samples), univariate logistic regression analyses was performed to calculate the odds ratio and corresponding 95% confidence intervals. Resistant rates was analysed with radar plots while Median and 75^th^ percentile resistance were evaluated with Boxplot analysis. Significance of resistance level of staphylococci strains was determined with chi-square and staphylococcal infectivity was calculated with multiple comparison using Kruskal-wallis test.

## Results

### Risk factor for staphylococcal infection and phenotypic resistance pattern

Staphylococcal infection was significant among the ages (p<0.05, OR[CI] = 0.021[0.545–1.914]) while higher prevalence rate of MSSA (53.0%) and MRSA (3.0%) infection were recorded among female and male subjects respectively. MSSA (37.9%) and MRSA (1.5%) infection rates were significant in wound infection as other clinical conditions presented by the subjects (p<0.05) while MSSA (42.2%) were observed in other conditions (eye, throat and endocervical infection) as shown in Table 1 and S1 Table.

**Table 1:** Univariate analysis of staphylococcal infections.

Characteristic	MSSA n(%)	MRSA n(%)	OR(CI)	P value
Age (yrs) (Median age: 36.5)	63(24.6)	3(1.2)	0.021(0.545–1.914)	0.004
**Gender**				
Female	35(53.0)	1(1.5)	1.021(0.374–1.785)	0.013
Male	31(47.0)	2(3.0)		
**Clinical samples**				
Otitis media	16(24.2)	1(1.5)		
Wound infection	25(37.9)	1(1.5)		
Purulent pus	12(18.2)	0(0.0)	0.434(0.569–4.183)	0.039
Aspirate effusions	13(19.7)	1(1.5)		
*Other infections	108(42.2))	0(0.0)		

(P<0.05 significant

*other infection include eye infection, throat and endocervical collections, n, number;%, percentage rate).

**Fig 1:**
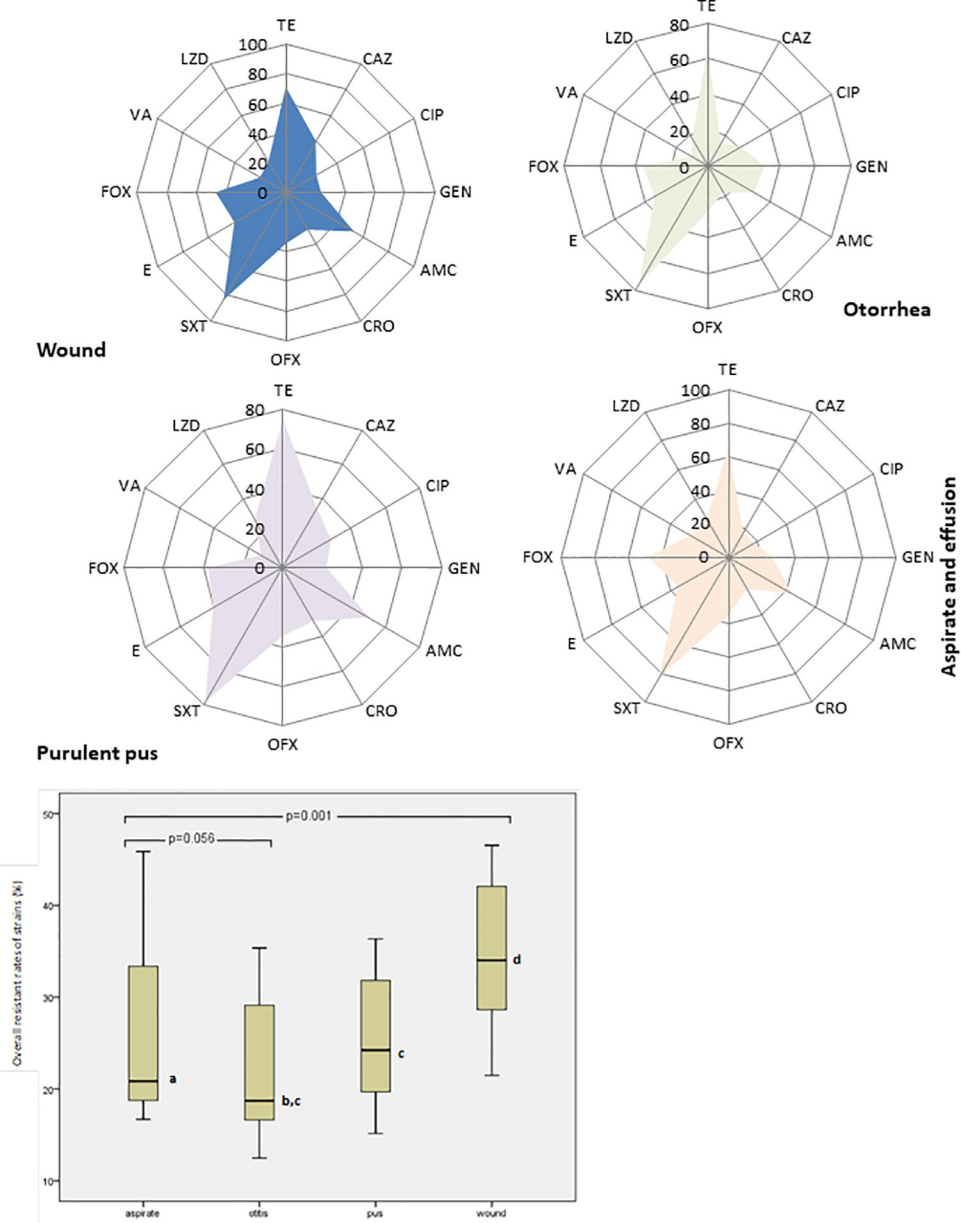
Radar plot of antibiotic resistance profile of *Staphylococus aureus* strains obtained from different infections and Box plot showing median distribution of antibiotic resistance pattern.

More than 52.5% resistant rates to tetracycline, sulphamethaxazole, gentamycin and amoxicillin were recorded among *S*. *aureus* from different sources as shown in Fig 1. The Box plot further reveal the overall antibiotic resistance rates of staphylococcal strains in different clinical conditions with significant estimated median resistance in all the presenting clinical disease conditions (p = 0.001) excluding susceptible strains isolated from eye, throat and endocervical samples but strains from aspirates and otitis had close median resistance rates (p = 0.056). Highest percentile (75^th^) and median resistance were observed in wound strains than others. Overall resistance rate of 78.8% to AMC at MIC_90_ (128μg/ml) was recorded for strains obtained from aspirates, while strains recovered from pus, ear and wound infections showed more than 30% resistance at MIC_50_ (8–16μg/ml). *S*. *aureus* strains (59.3%) recovered from pus and aspirate were resistant to CRO at MIC_90_ (64μg/ml) and MIC_50_ (8μg/ml), respectively. More than 40% of *S*. *aureus* strains obtained from aspirate had high resistance to TET (MIC_90_ and MIC_50_ at 64μg/ml), while strains recovered from pus were resistant to GN (MIC_90_ and MIC_50_ at 128μg/ml and 16μg//ml), respectively (Table 2).

**Table 2:** Phenotypic resistant of *S*. *aureus* strains from various infection sources to antibiotics.

Antibiotics Agents	Range	Break point of resistance	Wound (*n* = 26)		Ear (*n* = 18)		Pus (*n* = 13)		Aspirate (*n* = 9)		Percentage of resistance (%)
		MIC (μg/ml)									
			MIC_50_	MIC_90_	MIC_50_	MIC_90_	MIC_50_	MIC_90_	MIC_50_	MIC_90_	
**TE**	0.25–128	16	8	32	4	64	4	64	8	64	43.0
**CAZ**	0.1–64	4	4	64	4	32	16	64	2	32	36.5
**CIP**	0.12–16	4	4	16	1	64	2	64	1	32	38.9
**GEN**	0.03–2.0	1	2	16	1	64	2	128	16	64	40.2
**AMC**	0.25–64	16	2	32	2	32	1	32	8	128	78.8
**CRO**	0.1–64	4	8	32	2	32	2	64	4	64	59.3
**OFX**	0.12–64	4	4	16	1	64	1	32	8	32	35.6
**SXT**	0.5–128	32	4	32	16	128	16	128	16	64	41.7
**E**	0.5–64	32	1	16	4	16	4	64	16	128	34.0
**FOX**	0.1–64	4	2	16	2	32	8	128	8	64	46.5
**LZD**	0.1–64	2	1	16	1	32	4	64	2	32	34.2
**VA**	0.1–64	4	1	8	4	32	16	32	8	4	30.1

Notes: N, number of isolates; N, Number of samples; TE, Tetracycline; CAZ, Ceftazidime; CIP, Ciprofloxacin; GEN, Gentamycin; AMC, Amoxycillin-clavulanic acid; CRO, Cefuroxime; OFX, Ofloxacin; SXT, sulfamethoxazole; E, Erythromycin; FOX, fosfomycin; LZD, Linezolid; VA, Vancomycin, MIC; Minimum inhibitory concentration.

### Resistance relatedness of extra-intestinal *S*. *aureus* strains

**Fig 2:**
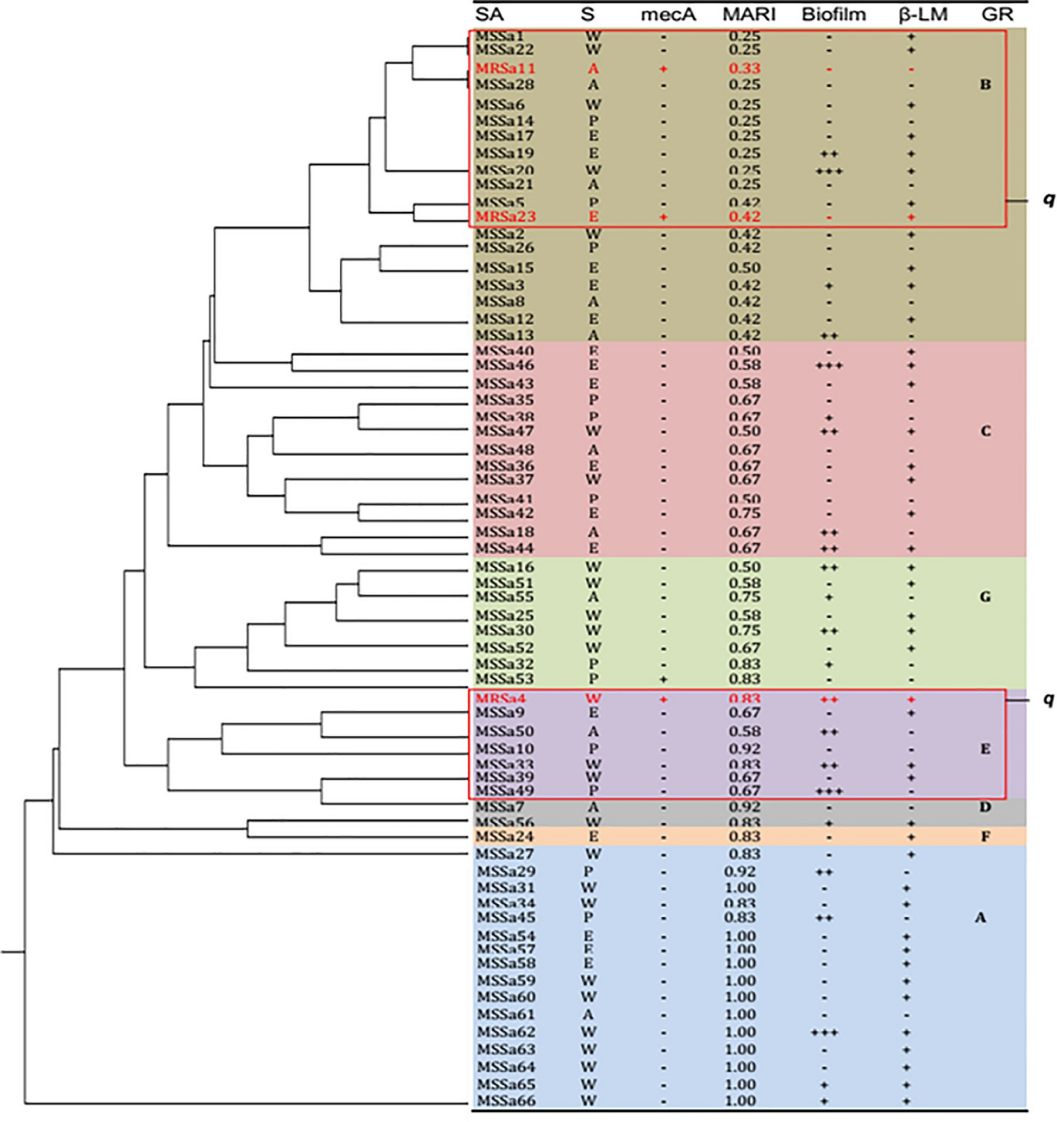
Antibiotic resistance relatedness of the recovered Staphylococci strains with high multi-antibiotic resistance index (MARI), biofilm and beta-lactamase production and mecA genotype.

Only three *S*. *aureus* strains recovered from aspirate, otitis media and wound expressed *mecA* gene (Fig 2), several strains clustered into group C with similar MARI of more than 0.50, characterized with biofilm and high beta-lactamase production. More than 0.83 MARI were observed among the strains that clustered into group A with high number of strains producing beta-lactamase and strong biofilm, but only one strain of MARI 0.92 clustered to Group D. In all, only 23/66 strains were biofilm producer but differed in level of production.

### Clonal diversity of identified spa types

**Fig 3:**
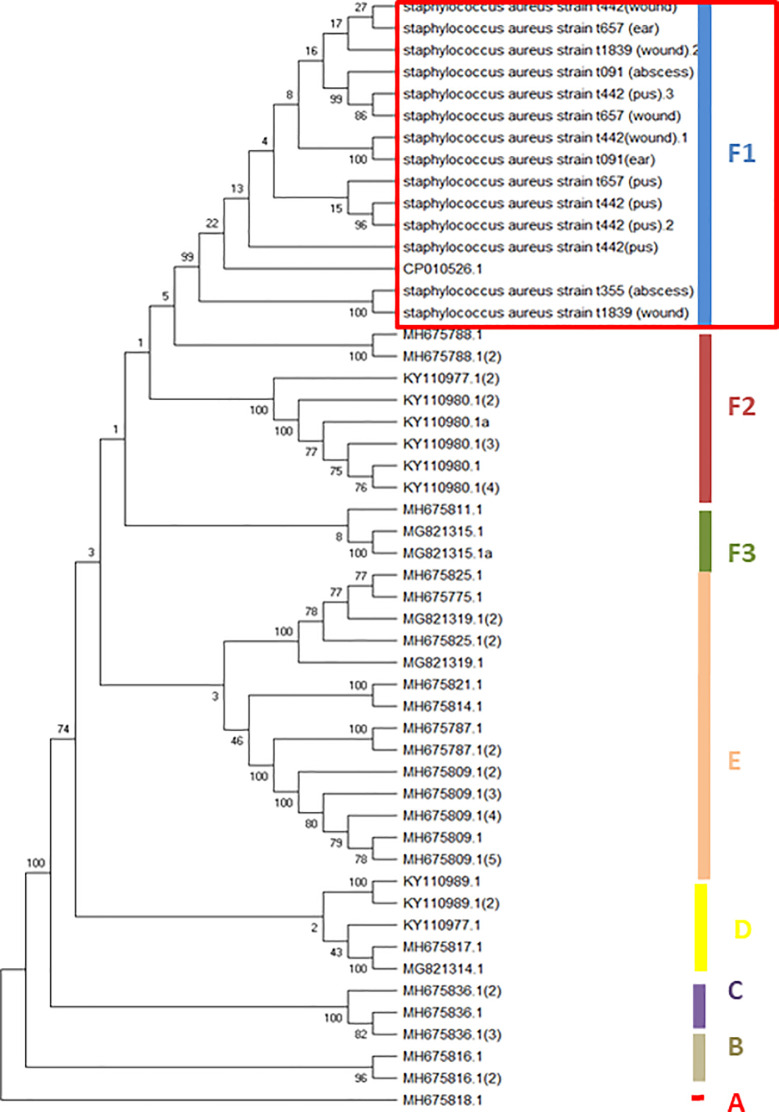
Neighbor-joining tree showing the phylo-diversity of Staphylococci characterized by heterogeneous *spa* types.

Heterogeneous *spa* types from extra-intestinal staphylococci strains clustered meta-spa types into six separate clades, of which *spa* t442 (from wound and pus), t657 (wound), t091 (otitis media) and t657 (otitis media and wound) clustered into clade F1 with other spa types from blood stream and soft tissue infection (red rectangular). High phylogenetic relatedness of *spa* sequences of livestock-associated *S*. *aureus* strains (bovine milk-MH675788.1, MG821315.1 and MH675814.1) clustered with the human strains (Fig 3).

### Encoded *agr* and focal dissemination

**Fig 4:**
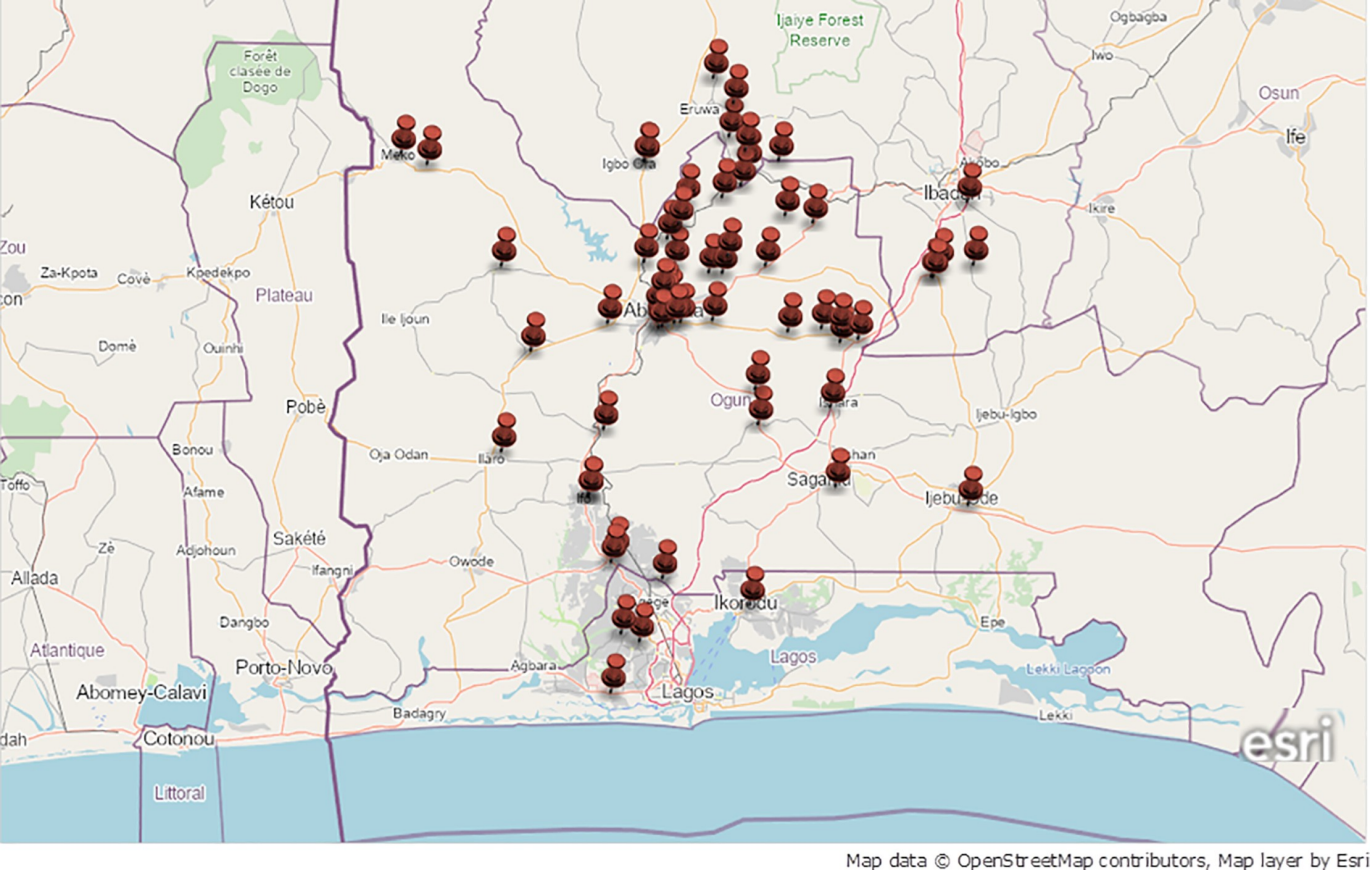
Geospatial mapping and focal dissemination of multi-antibiotic resistance *Staphylococcus aureus* pathotypes characterized with heterogenous spa genes distributed in various communities divided according to boundary marks in southwest Nigeria.

Clonal strains *pvl*+ MSSA-CC1 (4.6%) obtained from wound samples, of *spa* t1839, majorly encoded exfoliative toxin *(etD*, *etB)*, proteases *(aur*, *slpA sspB*, *sspE*, *sspP)* and resistant determinants; *bla (*beta lactamase repressor (inhibitor) and beta-lactamase regulatory protein); *fosB (*Metallothiol transferase); *sdrM (tet* efflux protein) and *Q2YUB3* (Multidrug resistance transporter) (Table 3), expressed *agr*I functionality. Obviously, 13.6% *pvl*+ MSSA belonging to clonal lineage CC5 from pus, wound and abscess harboured numerous heterogeneous *spa* types with functional *agr*II encoding enterotoxin *sea*, *sec*, *sed*, *sej*, *sel*, *ser*), leukocidins (*LukF-PV*, *lukD*, *lukE)* and proteases *(aur*, *slpA sspB*, *sspE*, *sspP)*. In addition, *agr*II was also recorded in 4.6% *pvl+* MRSA-CC7 strains of *spa* t091 characterized with *LukF-PV*, *lukD*, *lukE*, proteases *and aphA3*, (3,5-aminoglycoside phosphotransferase encoding neomycin/ kanamycin resistance); *sat (*Streptothricine-acetyltransferase); *tetK* (Tetracycline resistance markers); *msr (A) (*Macrolide efflux); and *mph(C)*, (lysylphosphatidyl-glycerol synthetase). Most MSSA strains were observed to be prevalent at urban communities showing focal dissemination to other nearest suburbs while identified MRSA was observed to be spreading together with other MSSA strains (Fig 4).

**Table 3:** Functional *agr*, clonal types and gene determinants in MSSA and MRSA *pvl* positive strains.

*Agr* types	Strains (%)	Sources	Clonal Complex	*spa* types	*Virulence determinants*	*Antibiotic resistance genes*
*agr*I	MSSA (4.6)	wound	CC1(ST772,ST573)	t1839	*sea*, *lukD*, *lukE*, *sak*, *chp*, *scn*, *etD*, *etB*, *aur*, *slpA sspA*, *sspB*,*sspP*	*Q2YUB3*, *fosB*, *sdrM*, *bla*, *dfrG*,*tetK*
*agr*II	MSSA (13.6)	Pus, wounds, abscess	CC5(ST5, ST73, ST492, ST1447)	t002, t010, t053, t067, t088, t179, t214, t242, t442, t509, t688, t1062, t1265, t6709	*Sea*, *sec*, *sed*, *sej*, *sel*, *ser*, *LukF-PV*, *lukD*, *lukE*, *scn*, *aur*, *splA sspA*, *sspB*,*sspP*	*fosB*, *msr (A)*, *bla mph(C)*,*aphA3*, *sat*,*fosB*, *sdrM*, *Q7A4X2*
*agr*II	MRSA (4.6)	wound	CC7(ST789)	t091	*lukD*, *lukE*, *sak*, *scn*, *aur*, *splA*, *slpE*, *sspA*, *sspB*,*sspP*	*bla*, *fosB*,*aacA-aphD*, *aphA3*,*sat*,*tetK*,*sdrM*, *ccrC*, *aacA-aphD*,*aphA3*,

Note: Enterotoxin genes (*sea*, *sec*, *sed*, *sej*, *sel*, *ser*); Leukocidins (*LukF-PV*, *lukD*, *lukE); exfoliative toxin (etD*, *etB); Proteases(aur*, *slpA)*, *bla (*beta lactamase repressor (inhibitor) and beta-lactamase regulatory protein); *fosB (*Metallothiol transferase); *aacA-aphD (*Bifunctional enzyme Aac/Aph; gentamicin, tobramycin resistance); *aphA3*, (3,5-aminoglycoside phosphotransferase, neo-/ kanamycin resistance); *sat (*Streptothricine-acetyltransferase); *tetK* (Tetracycline resistance markers); *sdrM (*Multidrug efflux protein, tetEfflux); *msr (A) (*Macrolide efflux); *mph(C) (*Probable lysylphosphatidyl-glycerol synthetase); *Q7A4X2* (Putative protein); *Q2YUB3* (Multidrug resistance transporter).

## Discussion

Continuous spread of staphylococcal infection in several communities is now becoming a threat to the populace and mostly the children. Methicillin susceptible *S*. *aureus* infections are now commonly observed among the children with high risk of sores, blood stream and scalded skin infections which are recorded due to low immunity, poor hygiene and possible transmission from staphylococci-carrier mothers [28]. Occupation and routine activities of many young adults and men (in population median age 36.5 years) could be considered a pre-disposing risk factor. Data relating subject occupation with staphylococcal infection was not available but high record of MRSA and MSSA detection in wound largely suggest stemming increase and spread of community-acquired staphylococcal infections [29]. Nosocomial staphylococcal infection could not be ruled out as hospital infection control could be compromised due to low hygiene and staff carriage of multi-antibiotic resistance staphylococci strains [30]. A significant low susceptibility was observed among the strains collection to ceftazidime, ciprofloxacin, amoxycillin-clavulanic acid and cefuroxime. Particularly strains from wound, ear, pus and aspirates showed a reflection of prolonged use and misuse of antibiotics in the treatment of staphylococcal infections. Continuous evolution and selective pressure of antibiotic resistance cannot be ruled out as a driven factor for the prevalence of resistant pathotypes across various population groups as evident with more than 40% resistance to tetracycline. Similar high level tetracycline and sulfamethoxazole resistance were already recorded in African *S*. *aureus* and animals strains [31,32].

The ability to treat multi-antibiotic resistant staphylococci strain characterized with biofilm is a challenging situation [33] and detection of different phylo-related strains expressing high level resistance with potential to produce both biofilm and beta-lactamase enzymes put the populace at great risk [34]. Antibiotic resistance relatedness of several MSSA showing observable *in-vitro* biofilm production reflects acute systemic infection severity and pathology that could progress to high morbidity [35,36], making MSSA-biofilm producing strains in soft tissue and skin infections difficult to treat [37]. High biofilm production in deep layer secretions in cases of septic wound, tissue abscess and purulent pus exudates could reduce drug penetration, inflammatory response and impairment of cellular immune activity [38]. In addition, strains with high MARI, beta-lactamase and high biofilm production are considered important pathotypes that needed to be designated for surveillance and assessment among diverse population at different localities. It is highly imperative to have periodic surveillance for these clusters with related resistance profile toward prevention of local sporadic outbreak and control of antibiotic misuse. However, unregulated prescription and abuse of antibiotics in several local communities in southwest Nigeria largely contribute to increase circulating resistant phylo-groups. Relative increase of resistant MRSA isolates to penicillin derivatives has been found to be associated with encoded *mecA* gene and beta-lactamase production which is a major factor to be considered towards achievable control of MRSA spread [39].

In addition, identification of heterogeneous *spa* types in extra-intestinal infections clearly showed high phylo-diverse *spa* strains clustering into various different clades. In spite of this strain-diversity, profound relatedness with other meta-spa types suggests high level dissemination of similar clonal groups [40]. This is a clear evidence of involvement of *spa* types in single or multiple staphylococcal infections having high substantial impact through localization and distribution in soft tissue for adaptation, colonization and pathogenesis thereby initiating severe infection [41,42].

Identified phylo-diverse MSSA from Nigerian communities indicates active transfer of clonal strains to other locations [43]. Detection of heterogeneous *spa* sequences from various skin and soft-tissue infections (wound, abscess and pus), is an evidence of genetic recombination of *spa* repeats from livestock-associated staphylococci particularly bovine milk [44]. This further establish animal to human transfer which is observed in most communities where animal husbandry is usually practice within and around the households. Consumption of unpasteurised bovine milk, poor milk wastes disposal and frequent human contact with udder during animal milking are observable predisposing risk factors to be considered as major sources and spread of diverse *spa* strains [44]. It is also important to note that reported multi-antibiotic resistant MRSA identities in this study could perpetuate severity with little or no therapeutic options. Scratches, pecking and bite on human skin by poultry, cattle and other livestock cannot be ruled out as major contributor to animal clonal strains found among this populace. It is imperative to investigate mechanism of animal transfer of *spa* types to human and high prevalence of these associated livestock *spa* types. It is also necessary to evaluate the emerging animal clonal *spa* types vis-a-vis animal husbandry and antibiotic residue in milk in order to safeguard the populace and drastically reduce dissemination and risk of contracting antibiotic resistant strains. Findings on animal related MRSA and MSSA *spa* types in humans, illustrates livestock involvement in continuous spread and distribution of Staphylococcal pathotypes in many communities. To control the prevalent, milk hygiene and animal waste management would enhance reduction in spread and skin infectivity particularly among children.

Resistant *S*. *aureus* encoding functional *agr* is known to have well-characterised operons controlling and regulating exfoliative toxin and protease genes in *pvl+* MSSA-CC1 strains which are in wound infections [45,46], would require continuous and strategic interventional approaches, door-to-door awareness program and routine MRSA and MSSA surveillance as important strategies for effective reduction of severe complications, morbidity, and occasional mortality. Predominant *agr*I and *agr*II in MSSA and occurrence of *agr*II in *pvl+* MRSA-CC7 clonally differ from *agr*III that were reported in Tunisia [47,48]. Expression of functional *agr*II in resistant *pvl*+ MSSA-CC5 and *pvl*+ MRSA-CC7 clones in pus, wound and abscess would further intensify invasiveness through action of enterotoxin genes (particularly *sea*, *sec*, *sed*, *and sej*), leukocidins (*LukF-PV*, *lukD/lukE)* and proteases *(aur*, *slpA sspB*, *sspE*, *sspP)*. Furthermore, bloodstream, skin and soft-tissue infections would be more severe in *agr* controlled staphylococcal diseases and could result in longer hospital stay, increase debilities and therapeutic failure. In rural and semi-urban settings with poor health facilities and hygiene awareness, dissemination of these resistant clonal pathotypes would exacerbate infection burden, mostly among the vulnerable elderly. Major limitations to the study were inadequate provision of demographic data, low retrieval of residence locations of the recruited subjects and their level of closeness to livestock around the households.

## Conclusion

Control of skin and soft tissue staphylococcal infections predominantly caused and spread by *agr* encoded *pvl+* MSSA-CC1 and *pvl+* MRSA-CC5 strains characterised with very high antibiotic resistance would require aggressive implementations of antibiotic stewardship, public health regulation, hygiene practice and extensive community health care intervention coupled with well-structured strategic infection control programs. Periodic geno-surveillance and investigation of multi-antibiotic resistant zoonotic MSSA and MRSA needed to be implemented concurrently with formulated health policy to prevent imminent outbreak of these clonal pathotypes.

## Supporting information

S1 Table Identified *Staphylococcus aureus* pathotypes with spa types obtained from different communities in southwest Nigeria. (DOCX)
