# Supplementary material for: Clonal diversity and spatial dissemination of multi-antibiotics resistant Staphylococcus aureus pathotypes in Southwest Nigeria
Source: PLoS One. 2021 Feb 23;16(2):e0247013. doi: 10.1371/journal.pone.0247013 (PMC7901740; doi:10.1371/journal.pone.0247013)
Supplement: S1 Table — (DOCX) [file pone.0247013.s001.docx]

Supplementary Table 1. Identified Staphylococcus aureus pathotypes with spa types obtained from different communities in southwest Nigeria.

| LOCATION | SOURCE | AGE | SEX | pvl | mecA | Spa typing | Strain |
| --- | --- | --- | --- | --- | --- | --- | --- |
| Abeokuta | Wound | 49 | M | - | - | t091 | Staph aureus |
| Abeokuta | Wound | 49 | M | - | - | - | Staph aureus |
| Odeda | Ear | 1 | F | - | - | - | Staph aureus |
| Abeokuta | Ear | 1 | F | - | - | - | Staph aureus |
| Abeokuta | Pus | 1 | M | - | - | t002, t010, t053, t067, t088, t179, t214, t242, t442, t509, t688, t1062 | Staph aureus |
| Abeokuta | Wound | 17 | M | - | - | - | Staph aureus |
| Abeokuta | Wound | 17 | M | - | - | - | Staph aureus |
| Odeda | Pus | 1 | M | - | - | - | Staph aureus |
| Odeda | Pus | 1 | M | - | - | - | Staph aureus |
| Abeokuta | Ear | 48 | F | - | - | - | Staph aureus |
| Ibadan | Wound | 21 | F | - | - | t442, t1839 | Staph aureus |
| Abeokuta | Pus | 9 | F | - | - | t442 | Staph aureus |
| Ibadan | abscess | 13 | M | + | - | t355 | Staph aureus |
| Abeokuta | Wound | 14 | F | - | - | t442, | Staph aureus |
| Sagamu | Abscess | 1 | M | - | - | - | Staph aureus |
| Abeokuta | Pus | 3 | F | + | - | t657 | Staph aureus |
| Abeokuta | Pus | 21 | F | - | - | t442 | Staph aureus |
| Lagos | Ear | 1 | M | - | + | - | Staph aureus |
| Abeokuta | Wound | 5 | F | - | - | - | Staph aureus |
| Abeokuta | Wound | 39 | M | + | - | + | Staph aureus |
| Abeokuta | Ear | 1 | M | + | - | + | Staph aureus |
| Lagos | Ear | 2 | M | - | - | t657, t002, t010, t053, t067, t088, t179, t214, t242, t442, t509, t688, t1062, t1265,t6709 | Staph aureus |
| Abeokuta | Ear | 2 | M | - | - | - | Staph aureus |
| Abeokuta | Ear | 2 | M | - | - | - | Staph aureus |
| Lagos | Pus | 62 | F | - | - | t442 | Staph aureus |
| Odeda | Abscess | 48 | F | - | + | t091 | Staph aureus |
| Abeokuta | Wound | 33 | F | - | - | + | Staph aureus |
| Ibadan | Wound | 23 | F | - | - | - | Staph aureus |
| Abeokuta | Wound | 23 | F | - | - | - | Staph aureus |
| Abeokuta | Pus | 21 | M | - | - | - | Staph aureus |
| Abeokuta | Pus | 21 | M | - | - | - | Staph aureus |
| Abeokuta | Wound | 50 | F | - | - | - | Staph aureus |
| Mowe | Wound | 13 | F | - | - | t442 | Staph aureus |
| Abeokuta | Abscess | 1 | M | - | - | - | Staph aureus |
| Abeokuta | Abscess | 1 | M | - | - | - | Staph aureus |
